# Investigation of *IGF1*, *IGF2BP2,* and *IGFBP3* variants with lymph node status and esophagogastric junction adenocarcinoma risk

**DOI:** 10.1002/jcb.27834

**Published:** 2018-10-18

**Authors:** Weifeng Tang, Shuchen Chen, Jun Liu, Chao Liu, Yafeng Wang, Mingqiang Kang

**Affiliations:** ^1^ Department of Cardiothoracic Surgery Affiliated People’s Hospital of Jiangsu University Zhenjiang Jiangsu China; ^2^ Department of Thoracic Surgery Fujian Medical University Union Hospital Fuzhou Fujian China; ^3^ Key Laboratory of Ministry of Education for Gastrointestinal Cancer, Fujian Medical University Fuzhou Fujian China; ^4^ Fujian Key Laboratory of Tumor Microbiology, Fujian Medical University Fuzhou Fujian China; ^5^ Central Lab, First Affiliated Hospital of Fujian Medical University Fuzhou Fujian China; ^6^ Department of Cardiology The People’s Hospital of Xishuangbanna Dai Autonomous Prefecture Jinghong Yunnan China

**Keywords:** adenocarcinoma, IGF1, IGF2BP2, IGFBP3, lymph node metastasis, polymorphism, susceptibility

## Abstract

Esophagogastric junction adenocarcinoma (EGJA) may be associated with obesity and overweight. Thus, any variant in energy metabolism–related gene may influence the development of EGJA. In this study, we recruited 720 EGJA cases and 1541 noncancer controls. We selected *IGF2BP2* rs4402960 G > T, rs1470579 A > C, *IGF1* rs5742612 A > G and *IGFBP3* rs3110697 G > A, rs2270628 C > T and rs6953668 G > A loci and assessed the relationship of these polymorphisms with lymph node status and susceptibility of EGJA. We found that *IGF2BP2* rs1470579 A > C and *IGFBP3* rs6953668 G > A polymorphisms were associated with the decreased risk of EGJA ( *IGF2BP2* rs1470579: CC vs AA: adjusted odds ratio [OR] = 0.65, 95% confidence interval [CI] = 0.43‐0.98, *P* = 0.041 and CC vs AA/AC: adjusted OR = 0.62, 95% CI = 0.41‐0.93, *P* = 0.021 and *IGFBP3* rs6953668: GA vs GG: adjusted OR = 0.66, 95% CI = 0.47‐0.93, *P* = 0.019 and GA/AA vs GG: adjusted OR = 0.68, 95% CI = 0.48‐0.95, *P* = 0.026). However, we also found that *IGF1* rs5742612 A > G polymorphism increased the risk of LNM among patients with EGJA (GG vs AA: adjusted OR = 1.88, 95% CI = 1.02‐3.46, *P* = 0.042 and GG vs AA/AG: adjusted OR = 1.92, 95% CI = 1.06‐3.47, *P* = 0.032). This study suggests that *IGF2BP2* rs1470579 A > C and *IGFBP3* rs6953668 G > A polymorphisms may decrease genetic susceptibility to EGJA in eastern Chinese Han population. In addition, our findings also indicate that *IGF1* rs5742612 A > G polymorphism may increase the susceptibility of LNM among patients with EGJA.

## 1 INTRODUCTION

In the past few decades, the incidence of esophagogastric junction adenocarcinoma (EGJA) has been increasing worldwide.^1^, ^2^ According to its anatomical region relative to the esophagogastric junction (EGJ), EGJA has been divided into three subtypes by the Siewert classification. Siewert type I and type III of EGJA are usually considered as esophageal and gastric cancer, respectively. Siewert type II malignancies are treated as “true” EGJA. However, the etiology and potential risk factor remain unclear. Recently, obesity and overweight have been known cancer risk factors. In addition, EGJA has been considered as an obesity and overweight‐related cancer.^3^, ^4^, ^5^ Thus, any variant and abnormal expression in energy metabolism gene may influence the development of EGJA.

Insulin‐like growth factor‐1 (IGF1), a growth hormone similar in molecular structure and function to insulin, may be implicated in growth during childhood and continue to have metabolism‐related influences in adults. IGF1 is generally produced by the liver. Most of the IGF1 bind to insulin‐like growth factor binding proteins (IGFBPs). IGFBP3 is the most abundant protein and binds to IGF1. It is found that the IGF signaling pathway plays an important role in some cancers.^6^ Gallagher et al^7^ have reported that patients with Laron syndrome have a decreased susceptibility of developing cancer. Dietary interventions and modifications may downregulate IGF1 activity and reduce the susceptibility of cancer by promoting increased glucagon activity.^8^ Recently, some case‐control studies have focused on the relationship of *IGFBP3* and *IGF1* single nucleotide polymorphisms (SNPs) with the risk of cancer.^9^, ^10^, ^11^ A previous case‐control study indicated that *IGFBP3* rs2270628 C > T was associated with an increased risk of ovarian cancer.^12^ Also, significant association with the survival of breast cancer in Chinese premenopausal women was identified for *IGFBP3* rs3110697 G > A.^13^ Liu et al^14^ reported that *IGFBP3* rs2270628 C > T and rs3110697 G > A SNPs were associated with a significantly decreased risk of esophageal squamous‐cell carcinoma (ESCC). In addition, some case‐control studies focused on the relationship of *IGF1* SNPs and gastric cancer.^15^, ^16^ *IGF1* rs5742612 A > G polymorphism was found to be associated with tumor response to chemotherapy in patients with advanced gastric cancer.^17^

Insulin‐like growth factor 2 mRNA‐binding protein 2 (IGF2BP2) is encoded by the *IGF2BP2* gene and acts as an RNA‐binding protein of IGF2 mRNA.^18^ Functions of IGF2BP2 are associated with insulin resistance, lipid metabolism, and tumorigenesis.^19^, ^20^ Dai et al^21^ reported that IGF2BP2 is a tumor promoter, which drives tumor proliferation through HMGA1 and mRNAs IGF2. Results of the previous case‐control study demonstrated that *IGF2BP2* rs4402960 G > T was involved in the risk of cancer.^22^, ^23^ In addition, Liu et al^24^ found that *IGF2BP2* variants might be an independent predictor of chemotherapeutic response in patients with metastatic gastric cancer.

However, the associations of *IGFBP3, IGF2BP2* and *IGF1* SNPs with EGJA risk were unknown. In this study, with an aim to explore the relationship of *IGF1*, *IGFBP3*, and *IGF2BP2* SNPs with the development of EGJA, *IGF2BP2* rs4402960 G > T, rs1470579 A > C, *IGF1* rs5742612 A > G and *IGFBP3* rs3110697 G > A, rs2270628 C > T and rs6953668 G > A loci were selected and genotyped in 720 EGJA cases and 1541 controls.

## 2 MATERIALS AND METHODS

### 2.1 Subjects

In this case‐control study, we examined 720 patients (188 female, 532 male, mean age 64.21 ± 8.82 years) with EGJA diagnosed according to gastroscope and pathology. Consenting patients with EGJA treated between January 2014 and May 2016 in the Fujian Medical University Cancer Hospital and Union Hospital were enrolled in this study. In addition, 440 patients with EGJA were included in this study from Affiliated People’s Hospital of Jiangsu University from November 2010 to November 2016. The patients with autoimmune disease history, prior chemoradiotherapy, and a history of another malignancy were excluded. All patients with EGJA were Asians from the east region of China. The noncancer controls were selected randomly from the population of the same region of China and consisted of healthy Asian 1541 subjects (404 female, 1137 male, mean age 64.30 ± 10.19 years). Each subject enrolled in this study answered a routine prestructured questionnaire, and height and weight were measured. Body mass index (BMI) ≥ 24 was accepted as the criterion for overweight and obesity.^25^, ^26^ The status of lymph node metastasis (LNM) was also collected. The study was approved by the ethics committee at Jiangsu University, Zhenjiang City, China, and a written informed consent was obtained from each participant.

### 2.2 DNA extraction and genotyping

The genomic DNA was carefully extracted from 2 mL of whole blood samples using a Promega Blood DNA Purification Kit (Promega, Madison, WI). *IGF2BP2* rs1470579 A > C, rs4402960 G > T, *IGF1* rs5742612 A > G and *IGFBP3* rs3110697 G > A, rs2270628 C > T and rs6953668 G > A polymporphisms were genotyped using SNPscan genotyping assays from Genesky Biotechologies Inc (Shanghai City, China).^27^, ^28^ Ninety DNA samples were selected randomly for quality control. The genotypes of *IGF2BP2* rs1470579 A > C, rs4402960 G > T, *IGF1* rs5742612 A > G and *IGFBP3* rs3110697 G > A, rs2270628 C > T and rs6953668 G > A SNPs were checked by another laboratory technicians. And the results were not changed.

### 2.3 Statistical analysis

Continuous variables were expressed as the mean ± standard deviation. The Student *t* test was applied to compare the differences between patients with EGJA and noncancer controls. Chi‐square (*χ* ^2^) or Fisher’s exact tests were used to compare categorical variables (eg, age, sex, weight, height, BMI, and genotype and allele frequencies) between EGJA groups and controls. SAS software (Version 9.4; Cary, NC) was used for data analysis. A *P* value less than 0.05 was considered statistically significant. Internet‐based software (http://ihg.gsf.de/cgi‐bin/hw/hwa1.pl) was harnessed to determine whether the distribution of genotype frequencies was according to Hardy‐Weinberg equilibrium (HWE).

## 3 RESULTS

### 3.1 Baseline characteristics

We list the clinical characteristics, selected risk factors, and demographics in Table 1. In our study, 720 patients with EGJA and 1541 noncancer controls were included. Table 1 shows that age and sex were well matched between the two groups (*P* = 0.826 and 0.958, respectively). The gene symbol, minor allele frequency (MAF), HWE, and genotyping successful ratio for *IGF2BP2* rs1470579 A > C, rs4402960 G > T, *IGF1* rs5742612 A > G and *IGFBP3* rs3110697 G > A, rs2270628 C > T and rs6953668 G > A SNPs are presented in Table 2.

**Table 1:** Distribution of selected demographic variables and risk factors in EGJA cases and controls

	Overall Cases (n = 720)	Overall Controls (n = 1,541)	
Variable	n(%)	n(%)	*P* ^a^
Age (years)	64.21 ± 8.82	64.30 ± 10.19	0.826
Age (years)			0.312
<64	327(45.42)	735(47.70)	
≥64	393(54.58)	806(52.30)	
Sex			0.958
Male	532(73.89)	1,137(73.78)	
Female	188(26.11)	404(26.22)	
Smoking status			**0.015**
Never	525(72.92)	1,196(77.61)	
Ever	195(27.08)	345(22.39)	
Alcohol use			**0.001**
Never	608(84.44)	1377(89.36)	
Ever	112(15.56)	164(10.64)	
Height (cm)	164.8( ± 7.28)	166.2( ± 7.21)	** <0.001**
Weight (kg)	61.98( ± 10.35)	65.94( ± 9.78)	** <0.001**
BMI (kg/m^2^)			
<24	476(66.11)	827(53.67)	** <0.001**
≥24	244(33.89)	714(46.33)	
Lymph node status			
Positive	424(58.89)		
Negative	296(41.11)		
AJCC TMN stage			
I + II	211(29.31)		
III + IV	509(70.69)		

Abbreviations: BMI: body mass index; AJCC: American Joint Committee on Cancer.

Bold values are statistically significant (*P* < 0.05).

^a^ Two‐sided *χ* ^2^ test and student t test.

**Table 2:** Primary information for *IGF2BP2* rs1470579 A > C, rs4402960 G > T, *IGF1* rs5742612 A > G and *IGFBP3* rs3110697 G > A, rs2270628 C > T and rs6953668 G > A polymorphisms

Gene	SNPs	MAF^a^ for Chinese population (http://gvs.gs.washington.edu/GVS147/)	MAF in our controls (n = 1541)	*P* value for HWE^b^ test in our controls	Genotyping value (%)
*IGF2BP2*	rs4402960 G > T	0.26	0.23	0.002	98.94
*IGF2BP2*	rs1470579 A > C	0.27	0.24	0.010	99.12
*IGF1*	rs5742612 A > G	0.29	0.29	0.604	99.20
*IGFBP3*	rs2270628 C > T	0.21	0.19	0.044	99.12
*IGFBP3*	rs3110697 G > A	0.23	0.27	0.170	99.16
*IGFBP3*	rs6953668 G > A	0.04	0.05	0.661	98.36

^a^ MAF: minor allele frequency.

^b^ HWE: Hardy‐Weinberg equilibrium.

### 3.2 Association of IGF2BP2 rs4402960 G > T, rs1470579 A > C, IGF1 rs5742612 A > G and IGFBP3 rs3110697 G > A, rs2270628 C > T and rs6953668 G > A polymorphisms with EGJA

The genotype distributions of *IGF2BP2* rs1470579 A > C, rs4402960 G > T, *IGF1* rs5742612 A > G and *IGFBP3* rs3110697 G > A, rs2270628 C > T and rs6953668 G > A SNPs are shown in Table 3. We found that rs1470579 A > C variant in the *IGF2BP2* gene was a protective factor for EGJA (CC vs AA: crude odds ratio [OR] = 0.66, 95% confidence interval [CI] = 0.44‐0.99, *P* = 0.045 and CC vs AA/AC: crude OR = 0.63, 95% CI = 0.42‐0.94, *P* = 0.023). When compared with the *IGFBP3* rs6953668 GG genotype, *IGFBP3* rs6953668 GA and GA/AA genotypes were also associated with the risk of EGJA (GA vs GG: crude OR = 0.66, 95% CI = 0.47‐0.93, *P* = 0.017 and GA/AA vs GG: crude OR = 0.68, 95% CI = 0.48‐0.95, *P* = 0.024). After adjustment for the included risk factors (eg, BMI, gender, sex, alcohol use, and smoking status) by logistic regression analysis, these observed findings were not altered (*IGF2BP2* rs1470579 A > C: CC vs AA: adjusted OR = 0.65, 95% CI = 0.43‐0.98, *P* = 0.041 and CC vs AA/AC: adjusted OR = 0.62, 95% CI = 0.41‐0.93, *P* = 0.021 and *IGFBP3* rs6953668: GA vs GG: adjusted OR = 0.66, 95% CI = 0.47‐0.93, *P* = 0.019 and GA/AA vs GG: adjusted OR = 0.68, 95% CI = 0.48‐0.95, *P* = 0.026 [Table 3]).

**Table 3:** Logistic regression analyses of association between *IGF2BP2* rs4402960 G > T, rs1470579 A > C, *IGF1* rs5742612 A > G and *IGFBP3* rs3110697 G > A, rs2270628 C > T and rs6953668 G > A polymorphisms and risk of EGJA

	Cases (n = 720)		Controls (n = 1,541)					
Genotype	n	%	n	%	Crude OR (95% CI)	*P*	Adjusted OR^a^ (95% CI)	*P*
*IGF2BP2* rs4402960 G > T								
GG	408	58.37	924	60.08	1.00		1.00	
GT	258	36.91	508	33.03	1.10 (0.91‐1.33)	0.334	1.09 (0.90‐1.31)	0.396
TT	33	4.72	106	6.89	0.67 (0.45‐1.01)	0.057	0.68 (0.45‐1.02)	0.061
GT + TT	291	41.63	614	39.92	1.07 (0.90‐1.29)	0.445	1.06 (0.89‐1.28)	0.507
GG + GT	666	95.28	1,432	93.11	1.00		1.00	
TT	33	4.72	106	6.89	0.67 (0.45‐1.00)	0.050	0.68 (0.45‐1.01)	0.057
T allele	324	23.18	720	23.41				
*IGF2BP2* rs1470579 A > C								
AA	388	55.19	902	58.65	1.00		1.00	
AC	283	40.26	527	34.27	1.20 (1.00‐1.45)	0.055	1.20 (1.00‐1.45)	0.054
CC	32	4.55	109	7.09	**0.66 (0.44‐0.99)**	**0.045**	**0.65 (0.43‐0.98)**	**0.041**
AC + CC	315	44.81	636	41.35	1.15 (0.96‐1.38)	0.125	1.15 (0.96‐1.38)	0.128
AA + AC	671	95.45	1,429	92.91	1.00		1.00	
CC	32	4.55	109	7.09	**0.63 (0.42‐0.94)**	**0.023**	**0.62 (0.41‐0.93)**	**0.021**
C allele	347	24.68	745	24.22				
*IGF1* rs5742612 A > G								
AA	337	47.80	774	50.33	1.00		1.00	
AG	309	43.83	640	41.64	1.07 (0.89‐1.28)	0.500	1.09 (0.90‐1.32)	0.364
GG	59	8.37	124	8.06	1.05 (0.75‐1.47)	0.774	1.08 (0.77‐1.52)	0.640
AG + GG	368	52.20	764	49.67	1.11 (0.93‐1.32)	0.267	1.13 (0.95‐1.36)	0.171
AA + AG	646	91.63	1,414	91.94	1.00		1.00	
GG	59	8.37	124	8.06	1.04 (0.75‐1.44)	0.804	1.06 (0.76‐1.47)	0.727
G allele	427	30.28	888	28.87				
*IGFBP3* rs2270628 C > T								
CC	454	64.58	1,024	66.58	1.00		1.00	
CT	224	31.86	447	29.06	1.09 (0.90‐1.33)	0.371	1.09 (0.89‐1.32)	0.415
TT	25	3.56	67	4.36	0.81 (0.51‐1.31)	0.392	0.82 (0.51‐1.32)	0.420
CT + TT	249	35.42	514	33.42	1.09 (0.91‐1.32)	0.354	1.09 (0.90‐1.31)	0.393
CC + CT	678	96.44	1,471	95.64	1.00		1.00	
TT	25	3.56	67	4.36	0.81 (0.51‐1.29)	0.377	0.82 (0.51‐1.32)	0.410
T allele	274	19.49	581	18.89				
*IGFBP3* rs3110697 G > A								
GG	382	54.26	840	54.62	1.00		1.00	
GA	280	39.77	579	37.65	1.02 (0.85‐1.23)	0.800	1.03 (0.85‐1.24)	0.758
AA	42	5.97	119	7.74	0.75 (0.52‐1.08)	0.125	0.75 (0.52‐1.10)	0.137
GA + AA	322	45.74	698	45.38	1.01 (0.85‐1.21)	0.876	1.02 (0.85‐1.22)	0.837
GG + GA	662	94.03	1,419	92.26	1.00		1.00	
AA	42	5.97	119	7.74	0.76 (0.53‐1.09)	0.133	0.76 (0.53‐1.10)	0.142
A allele	249	25.85	817	26.56				
*IGFBP3* rs6953668 G > A								
GG	643	93.19	1,384	90.22	1.00		1.00	
GA	47	6.81	147	9.58	**0.66 (0.47‐0.93)**	**0.017**	**0.66 (0.47‐0.93)**	**0.019**
AA	0	0	3	0.20	‐	‐	‐	‐
GA + AA	47	6.81	150	9.78	**0.68 (0.48‐0.95)**	**0.024**	**0.68 (0.48‐0.95)**	**0.026**
GG + GA	690	100.00	1,531	99.80	1.00		1.00	
AA	0	0	3	0.20	‐	‐	‐	‐
A allele	47	3.41	153	4.99				

Abbreviations: BMI, body mass index; CI, confidence interval; OR, odds ratio.

Bold values are statistically significant (*P* < 0.05).

^a^ Adjusted for age, sex, BMI, alcohol use and smoking status.

However, we found that *IGF2BP2* rs4402960 G > T, *IGF1* rs5742612 A > G and *IGFBP3* rs3110697 G > A, rs2270628 C > T variants might be not associated with the development of EGJA (Table 3).

### 3.3 Association of IGF2BP2 rs4402960 G > T, rs1470579 A > C, IGF1 rs5742612 A > G and IGFBP3 rs3110697 G > A, rs2270628 C > T and rs6953668 G > A polymorphisms with Lymph node status in EGJA patients

As shown in Table 4, we found that *IGF1* rs5742612 A > G polymorphism had a tendency of increased risk to LNM among EGJA patients (GG vs AA: crude OR = 1.77, 95% CI = 0.97‐3.23, *P* = 0.063 and GG vs AA/AG: crude OR = 1.80, 95% CI = 1.00‐3.22, *P* = 0.050). After adjustment for BMI, gender, sex, alcohol use, and smoking status, this association was more significant (GG vs AA: adjusted OR = 1.88, 95% CI = 1.02‐3.46, *P* = 0.042 and GG vs AA/AG: adjusted OR = 1.92, 95% CI = 1.06‐3.47, *P* = 0.032).

**Table 4:** Logistic regression analyses of correlation between *IGF2BP2* rs4402960 G > T, 1470579 A > C, *IGF1* rs5742612 A > G and *IGFBP3* rs3110697 G > A, rs2270628 C > T and rs6953668 G > A SNPs and lymph node status in EGJA patients

	Positive (n = 424)		Negative (n = 296)					
Genotype	n	%	n	%	Crude OR (95% CI)	*P*	Adjusted OR^a^ (95% CI)	*P*
*IGF2BP2* rs4402960 G > T								
GG	238	57.49	170	59.65	1.00		1.00	
GT	158	38.16	100	35.09	1.15 (0.84‐1.58)	0.376	1.17 (0.85‐1.61)	0.338
TT	18	4.35	15	5.26	0.88 (0.43‐1.78)	0.715	0.91 (0.45‐1.87)	0.800
GT + TT	176	42.51	115	40.35	1.09 (0.81‐1.49)	0.569	1.11 (0.81‐1.51)	0.527
GG + GT	396	95.65	270	94.74	1.00		1.00	
TT	18	4.35	15	5.26	0.82 (0.41‐1.65)	0.576	0.84 (0.41‐1.70)	0.628
*IGF2BP2* 1470579 A > C								
AA	225	54.35	163	56.40	1.00		1.00	
AC	171	41.30	112	38.75	1.10 (0.81‐1.51)	0.529	1.11 (0.82‐1.52)	0.499
CC	18	4.35	14	4.84	0.93 (0.45‐1.92)	0.845	0.96 (0.46‐1.99)	0.907
AC + CC	189	45.65	126	43.60	1.09 (0.80‐1.47)	0.591	1.09 (0.80‐1.48)	0.585
AA + AC	396	95.65	275	95.16	1.00		1.00	
CC	18	4.35	14	4.84	0.89 (0.44‐1.83)	0.756	0.91 (0.44‐1.87)	0.800
*IGF1* rs5742612 A > G								
AA	197	47.36	140	48.44	1.00		1.00	
AG	177	42.55	132	45.67	0.96 (0.71‐1.31)	0.804	0.94 (0.68‐1.28)	0.673
GG	42	10.10	17	5.88	1.77 (0.97‐3.23)	0.063	**1.88 (1.02‐3.46)**	**0.042**
AG + GG	219	52.64	149	51.56	1.05 (0.77‐1.41)	0.776	1.02 (0.76‐1.39)	0.882
AA + AG	374	89.90	272	94.12	1.00		1.00	
GG	42	10.10	17	5.88	1.80 (1.00‐3.22)	0.050	**1.92 (1.06‐3.47)**	**0.032**
*IGFBP3* rs2270628 C > T								
CC	273	65.94	181	62.63	1.00		1.00	
CT	130	31.40	94	32.53	0.92 (0.67‐1.27)	0.607	0.95 (0.68‐1.31)	0.734
TT	11	2.66	14	4.84	0.52 (0.23‐1.17)	0.116	0.52 (0.23‐1.17)	0.114
CT + TT	141	34.06	108	37.37	0.87 (0.63‐1.18)	0.366	0.88 (0.64‐1.21)	0.429
CC + CT	403	97.34	275	95.16	1.00		1.00	
TT	11	2.66	14	4.84	0.54 (0.24‐1.20)	0.129	0.53 (0.23‐1.19)	0.121
*IGFBP3* rs3110697 G > A								
GG	221	53.13	161	55.90	1.00		1.00	
GA	168	40.38	112	38.89	1.11 (0.81‐1.51)	0.522	1.11 (0.81‐1.52)	0.519
AA	27	6.49	15	5.21	1.33 (0.69‐2.58)	0.400	1.46 (0.75‐2.85)	0.268
GA + AA	195	46.88	127	44.10	1.12 (0.83‐1.51)	0.467	1.13 (0.83‐1.53)	0.446
GG + GA	389	93.51	273	94.79	1.00		1.00	
AA	27	6.49	15	5.21	1.26 (0.66‐2.42)	0.481	1.38 (0.72‐2.66)	0.335
*IGFBP3* rs6953668 G > A								
GG	378	93.10	265	93.31	1.00		1.00	
GA	28	6.90	19	6.69	1.03 (0.56‐1.88)	0.922	1.07 (0.58‐1.96)	0.825
AA	0	0.00	0	0.00	‐	‐	‐	‐
GA + AA	28	6.90	19	6.69	1.03 (0.56‐1.88)	0.922	1.07 (0.58‐1.96)	0.825
GG + GA	406	100.00	284	100.00	1.00		1.00	
AA	0	0.00	0	0.00	‐	‐	‐	‐

^a^ Adjusted for age, sex, smoking, alcohol use and BMI status.

## 4 DISCUSSION

The incidence of EGJA is increasing worldwide. The etiology of EGJA may be very complicated. Recently, some publications reported that obesity and overweight were associated with the development of EGJA.^3^, ^4^, ^5^ Thus, the variants in energy metabolism–related gene may influence the susceptibility of EGJA. In this study, we explored the relationship of *IGF2BP2* rs4402960 G > T, rs1470579 A > C, *IGF1* rs5742612 A > G and *IGFBP3* rs3110697 G > A, rs2270628 C > T and rs6953668 G > A SNPs with the development of EGJA in 2261 subjects. We found that *IGF2BP2* rs1470579 A > C and *IGFBP3* rs6953668 G > A polymorphisms might be protective factors for EGJA. However, we identified that *IGF1* rs5742612 A > G polymorphism had an increased risk to LNM among EGJA patients.

*IGF2BP2* rs1470579 A > C polymorphism is located on intron 2. Recently, a meta‐analysis study reported that CC carriers of rs1470579 conferred risk to type 2 diabetes mellitus (T2DM) than *IGF2BP2* rs1470579 CA/AA carriers.^29^ Several case‐control studies assessed the potential association of *IGF2BP2* rs1470579 A > C variants with T2DM susceptibility and therapeutic efficacy in the Chinese population.^30^, ^31^ In these studies, *IGF2BP2* rs1470579 A > C polymorphism were found to be associated with T2DM risk, and this polymorphism may influence the therapeutic efficacy of some oral antidiabetic agents in patients with T2DM.^30^, ^31^ It is found that some variants in energy metabolism–related gene may influence the development of cancer.^22^, ^23^, ^32^ In the current study, we first explored the association of *IGF2BP2* rs1470579 A > C polymorphism with the risk of EGJA. It was found that the rs1470579 CC genotype of *IGF2BP2* gene might be a protective factor for the development of EGJA.

IGFBP‐3, a common IGF binding protein, has highly conserved structures and binds IGF‐1 and IGF‐2 with high affinity. Based on the functional studies, it is believed that IGFBP‐3 may be acting as a low‐penetrance tumor suppressor.^33^ Recently, some case‐control studies focused on the relationship between *IGFBP3* variants and cancer risk. Liu et al^14^ reported that *IGFBP3* rs2270628 C > T and rs3110697 G > A variants significantly decreased the risk of ESCC in Chinese Han population. However, in this study, we found that *IGFBP3* rs2270628 C > T and rs3110697 G > A SNPs were not associated with the risk of EGJA in the Chinese population. *IGFBP3* rs6953668 G > A polymorphism is located on intron. Verheus et al^34^ studied the relationship between *IGFBP3* rs6953668 G > A polymorphism and mammographic density. And they found a null association. However, we identified that *IGFBP3* rs6953668 G > A polymorphism may decrease the risk of EGJA. The current study did not assess the role of this SNP in regulating the expression of the IGFBP3 protein in tissue of patients with EGJA. In the future, a functional study is necessary to be performed.

Several case‐control studies focused on the relationship of *IGF1* rs5742612 A > G polymorphism with gastrointestinal cancer.^35^, ^36^ The results of these studies indicated that *IGF1* rs5742612 A > G polymorphism might be not associated with the risk of gastrointestinal cancer. In the current study, we found that *IGF1* rs5742612 A > G variants might be not associated with the development of EGJA. Our findings were similar to those studies mentioned above.

A previous study indicated that IGF‐1 and IGF‐1R are upregulated in tissue of non–small‐cell lung cancer (NSCLC), and expression of those factors was associated with the progression and prognosis of NSCLC.^37^ In addition, it was found that IGF‐1 may induce lymphangiogenesis and facilitates lymphatic metastasis,^38^ and be associated with larger tumor size, local LNM, and worse prognosis in cancers.^39^, ^40^ Oh et al^17^ reported that *IGF1* rs5742612 A > G polymorphism was significantly associated with tumor response to patients with gastric cancer treated with 5‐fluorouracil, leucovorin, and oxaliplatin. In this study, we found that *IGF1* rs5742612 A > G polymorphism might increase the risk of LNM among patients with EGJA. To our knowledge, this is the first study to confirm the relationship between *IGF1* rs5742612 A > G polymorphism and the risk of LNM. Wang et al^41^ reported that the G allele of rs5742612 was found to be associated with decreased insulin sensitivity and increased insulin secretion. In addition, insulin levels were found to be correlated with LNM risk in both premenopausal and postmenopausal women with endometrial cancer.^42^ In view of these findings, it is suggested that *IGF1* rs5742612 A > G polymorphism may increase insulin secretion and induce lymphangiogenesis and facilitates lymphatic metastasis. Thus, this SNP may be implicated in the development of EGJA.

In this study, some potential limitations should be addressed. First, the included patients with EGJA were limited, which may restrict to draw a strong conclusion. Secondly, only five SNPs were selected and genotyped; the coverage might be insufficient. In the future, for practical reasons, a fine‐mapping study is needed to extensively assess the correlation of these genes variants with the development of EGJA. Thirdly, in the current study, the information on other risk factors was lacking. A further analysis on the relationship between these loci and environmental characteristic was not performed. Finally, a functional study was not carried out to further explain the potential role of these SNPs.

In summary, this study suggests that *IGF2BP2* rs1470579 A > C and *IGFBP3* rs6953668 G > A polymorphisms may be associated with genetic susceptibility to EGJA in eastern Chinese Han population. In addition, our findings also demonstrate that *IGF1* rs5742612 A > G polymorphism may increase the risk of LNM among patients with EGJA.

## CONFLICTS OF INTEREST

The authors have no potential financial conflicts of interest.

## FUNDING

This study was supported in part by General Project of Health Development Planning Commission in Jiangsu Province (Z2017021), Young and Middle‐aged Talent Training Project of Health Development Planning Commission in Fujian Province (2016‐ZQN‐25), Program for New Century Excellent Talents in Fujian Province University (NCETFJ‐2017B015) and Joint Funds for the Innovation of Science and Technology, Fujian Province (2017Y9099).
